# AI in the Health Sector: Systematic Review of Key Skills for Future Health Professionals

**DOI:** 10.2196/58161

**Published:** 2025-02-05

**Authors:** Javier Gazquez-Garcia, Carlos Luis Sánchez-Bocanegra, Jose Luis Sevillano

**Affiliations:** 1 Servicio Andaluz de Salud, Distrito Sanitario Almeria, Almeria, Spain; 2 Faculty of Health Sciences, Universidad Oberta de Catalunya (UOC), Barcelona, Spain; 3 Universidad de Sevilla, ETS Ingenieria Informatica, Avda Reina Mercedes s/n, Sevilla, 41012, Spain, 34954556142

**Keywords:** artificial intelligence, healthcare competencies, systematic review, healthcare education, AI regulation

## Abstract

**Background:**

Technological advancements have significantly reshaped health care, introducing digital solutions that enhance diagnostics and patient care. Artificial intelligence (AI) stands out, offering unprecedented capabilities in data analysis, diagnostic support, and personalized medicine. However, effectively integrating AI into health care necessitates specialized competencies among professionals, an area still in its infancy in terms of comprehensive literature and formalized training programs.

**Objective:**

This systematic review aims to consolidate the essential skills and knowledge health care professionals need to integrate AI into their clinical practice effectively, according to the published literature.

**Methods:**

We conducted a systematic review, across databases PubMed, Scopus, and Web of Science, of peer-reviewed literature that directly explored the required skills for health care professionals to integrate AI into their practice, published in English or Spanish from 2018 onward. Studies that did not refer to specific skills or training in digital health were not included, discarding those that did not directly contribute to understanding the competencies necessary to integrate AI into health care practice. Bias in the examined works was evaluated following Cochrane’s domain-based recommendations.

**Results:**

The initial database search yielded a total of 2457 articles. After deleting duplicates and screening titles and abstracts, 37 articles were selected for full-text review. Out of these, only 7 met all the inclusion criteria for this systematic review. The review identified a diverse range of skills and competencies, that we categorized into 14 key areas classified based on their frequency of appearance in the selected studies, including AI fundamentals, data analytics and management, and ethical considerations.

**Conclusions:**

Despite the broadening of search criteria to capture the evolving nature of AI in health care, the review underscores a significant gap in focused studies on the required competencies. Moreover, the review highlights the critical role of regulatory bodies such as the US Food and Drug Administration in facilitating the adoption of AI technologies by establishing trust and standardizing algorithms. Key areas were identified for developing competencies among health care professionals for the implementation of AI, including: AI fundamentals knowledge (more focused on assessing the accuracy, reliability, and validity of AI algorithms than on more technical abilities such as programming or mathematics), data analysis skills (including data acquisition, cleaning, visualization, management, and governance), and ethical and legal considerations. In an AI-enhanced health care landscape, the ability to humanize patient care through effective communication is paramount. This balance ensures that while AI streamlines tasks and potentially increases patient interaction time, health care professionals maintain a focus on compassionate care, thereby leveraging AI to enhance, rather than detract from, the patient experience.

## Introduction

Technological advancements have transformed health care, improving diagnostics and patient care through solutions such as diagnostic support systems and telemedicine. These technologies reduce costs and enhance care quality, but their adoption faces challenges, particularly in terms of infrastructure, training, and education [1,2]. To address these challenges, the European Commission’s DigComp framework [3] outlines key competencies needed to adapt to new technologies, with a particularly challenging issue being the integration of artificial intelligence (AI) into health care.

AI systems use complex algorithms to analyze large datasets, identify patterns, and improve decision-making [4]. AI classification is multifaceted. The European Commission distinguishes between AI software, such as virtual assistants, and AI embedded in physical devices, such as robots [3]. Alternatively, Russell and Norvig’s [5] taxonomy assesses AI based on cognitive and behavioral capabilities, differentiating systems that emulate human or rational thought and action.

Each AI approach is designed to refine its problem-solving abilities, whether by mimicking human behavior or optimizing logical decisions [4]. AI improves clinical decision-making by addressing human limitations in data processing, supporting evidence-based practices through technologies such as machine learning and deep learning [6]. Various AI applications, such as image processing and convolutional neural networks, enhance clinical outcomes [6].

While human interaction remains central to health care, AI mitigates cognitive biases and provides faster, more precise outcomes [7]. For example, AI can process millions of medical images far faster than a human radiologist, improving accuracy through continual learning [6].

The integration of AI into health care is not just a technological advancement but a profound transformation of the operational, cultural, and ethical frameworks of health care organizations. This shift requires professionals to develop specialized knowledge in AI disciplines, such as machine learning, deep learning, and natural language processing, to use these tools effectively and ethically [8].

AI’s integration demands expertise and regulatory oversight to ensure ethical use, bias mitigation, and privacy protection [9]. The US Food and Drug Administration is exploring regulatory frameworks for AI-based algorithms in medicine, though a definitive process has yet to be established [10]. A recent study in *JAMA Ophthalmology* [11,12] highlighted the risks of AI misuse, including sophisticated models such as ChatGPT. Professionals are essential in identifying data falsifications that may not be evident to untrained individuals [12].

Addressing these challenges requires a concerted effort to enhance AI literacy and redesign clinical processes, fostering synergy between human judgment and AI-augmented decision-making [13,14]. This transition demands rigorous training in technical, procedural, and collaborative skills to ensure health care professionals can effectively integrate AI into practice, enabling them to adapt to the evolving technological landscape and improve clinical practice [15].

Numerous studies have explored the integration of AI in medical education, demonstrating its potential to enhance practical skills and personalize the learning experience for students. However, most of these reviews focus on the application of AI to improve medical education [16], which examines the use of various AI methods to enhance training in different medical domains.

Some studies [17] propose that training health care professionals in AI requires specialized roles, such as health information management professionals, to manage and adapt AI technologies in clinical settings. This approach ensures that AI integration occurs safely and efficiently, considering data quality, ethical, and legal aspects.

A review of how training programs for health care professionals deal with AI shows both the relatively low number of programs available and their significant limitations [18]. The authors recommend future curricula be designed with AI-related core competencies in mind.

This review aims to identify and highlight the critical competencies necessary to guide the development of educational programs designed to optimize the use of AI in clinical settings, thus addressing a growing need at the intersection of medicine and technology. By analyzing and synthesizing the existing literature on AI training for health care professionals, our goal is to provide a comprehensive framework that informs continuous education and specialized training in AI, ensuring the safe and effective implementation of these advanced technologies in daily clinical practice.

The integration of AI into health care presents several critical challenges. This study aims to consolidate and articulate the specific skills and knowledge required for health care professionals to effectively implement AI in routine clinical practice. Our research question is as follows: “In healthcare professionals, what specific skills and competencies are necessary for the effective implementation and use of AI technologies in daily clinical practice compared to their current skill set?” This question focuses on identifying and defining the critical competencies required for health care professionals to effectively use AI, providing a solid foundation for the development of educational and training programs in this emerging field.

## Methods

### Study Design

In conducting a systematic review between November and December 2023, we adhered to the PRISMA (Preferred Reporting Items for Systematic Reviews and Meta-Analyses) guidelines [19] (Multimedia Appendix 1 and Checklist 1).

### Data Sources and Search Strategy

Comprehensive searches were performed across databases including PubMed, Scopus, and Web of Science, using Health Science Descriptors and Medical Subject Headings descriptors pertinent to AI in health care and associated skills. The search strategy was refined using Boolean and truncation operators. The search queries included combinations such as (“Artificial Intelligence” OR “AI”) AND “Healthcare Professionals” AND (“Skills” OR “Competencies” OR “Education”), (“Artificial Intelligence” OR “AI”) AND (“data analysis” OR “ethical considerations”).

### Inclusion and Exclusion Criteria

We established specific selection criteria, prioritizing peer-reviewed original articles, review articles, editorials, and commentaries that directly explored the required skills for health care professionals to integrate AI into their practice. Regarding the inclusion criteria, studies were considered if they: were published in English or Spanish, taking advantage of the linguistic accessibility for the research team in order to reflect the possible specific characteristics of the Spanish-speaking community; published from 2018 onward, to ensure the relevance and timeliness of the research given the rapid advancements in AI technologies in recent years; focused on the necessary skills for the effective use of AI by health care professionals, encompassing both technical and management competencies; and included aspects related to training in digital health, highlighting the importance of specific training in the use of emerging technologies.

Conversely, studies were excluded if they did not meet these criteria: studies written in languages other than English or Spanish, as they could not be accurately analyzed by the research team; studies published before 2018, to focus on the most recent trends in AI in health care; studies not specifically related to the skills or needs of health care professionals for the use of AI tools, excluding research that did not directly address this focus; and studies that did not refer to specific skills or training in digital health, discarding those that did not directly contribute to understanding the competencies necessary to integrate AI into health care practice.

This selection methodology was designed to identify studies that provided significant evidence on the key competencies health care professionals need to develop for effectively integrating AI into their clinical practice, thus, ensuring that the systematic review focused on research offering practical and applicable insights.

### Data Extraction and Study Quality Assessment

The search strategy was developed iteratively to optimize the retrieval of relevant studies. An initial search used the aforementioned databases and descriptors. Titles and abstracts underwent rigorous review for relevance by 2 independent researchers (JG-G and CLS-B), with duplicates removed and articles not meeting inclusion criteria or fitting exclusion criteria discarded. Mendeley (Elsevier Ltd) served as the reference management tool, not involved in the data extraction process.

Subsequently, full-text articles were retrieved for an in-depth content review based on the inclusion criteria specified earlier. However, 3 articles were not retrievable despite repeated efforts. Two articles were inaccessible due to subscription restrictions, and attempts to obtain them via interlibrary loans were unsuccessful. The third article had a broken link, and the authors were unresponsive after multiple contact attempts. These articles have been documented in the “reports not retrieved” section of the PRISMA flow diagram in the Results section.

To assure methodological integrity, discrepancies were resolved through consultation with a third researcher (JLS), reaching consensus on study admissibility. The screening process was manual, without the use of automation tools, and each study was carefully evaluated. Mendeley was used again for reference management, without affecting the data extraction process. The PRISMA flow diagram is provided in the Results section.

Data were extracted and synthesized from eligible studies, encompassing details such as authors, publication year, study design, identified skills, and methodological quality assessment. Tables served as the primary method for tabulating and visually presenting the results and their synthesis.

The data search spanned all pertinent dimensions of these outcomes, including variables relevant to the analyzed studies. These variables covered aspects such as humanization, social skills, and participants’ AI usage experience, offering a comprehensive perspective on the competencies necessary for integrating AI into health care practices.

The GRADE (Grading of Recommendations Assessment, Development, and Evaluation) framework [20] was applied to assess the evidence quality of the included works, considering the quality of studies, result consistency, imprecision, potential bias, and other pertinent factors. Mixed methods or qualitative studies were appraised using the mixed methods appraisal tool [21].

Bias in the examined works was evaluated following Cochrane’s domain-based recommendations [22], considering five types of bias, each with its domains. Bias risk was independently assessed by at least 2 reviewers, with discrepancies resolved via discussion or consultation with a third reviewer when necessary.

### Synthesis Method

The research question guided the synthesis groupings, focusing the analysis on the skills and competencies necessary for the effective implementation of AI technologies by health care professionals. No standardization metric was applied. The synthesis method involved extracting relevant sections of the studies related to the identified skills and competencies. Overall, the risk of bias assessment of the studies showed no critical results. Consequently, the included studies were synthesized with equal weight.

The categorization of skills and competencies was based on a qualitative synthesis approach, where 2 independent reviewers extracted and coded data from each study. The categories were developed iteratively by grouping competencies that were conceptually aligned. The final domains were determined based on the frequency with which competencies appeared across the studies, with higher-frequency competencies categorized as key domains. For example, “AI fundamentals” and “ethical and legal considerations” were identified as critical due to their frequent mention in the selected studies, whereas others such as “data governance” and “programming” appeared less often but were still considered relevant.

Study design was not restricted, so a meta-analysis was not performed due to the heterogeneity of the studies and the differing amounts and qualities of information reported on skills and competencies. The reported effectiveness of the competencies was synthesized based on each study’s findings. The identified competencies are presented in the results section. Tables and figures aggregate information about this study’s characteristics and focal areas of this review (skills and competencies).

This review aimed to identify and delineate the skills and competencies essential for health care professionals to use AI in their clinical practices effectively. The objective encompassed various outcome domains, not limited to interpreting results from machine learning models, managing AI biases, ethical considerations in AI-assisted decision-making, and the technical skills required for effective AI tool use.

## Results

### Overview

**Figure 1.**
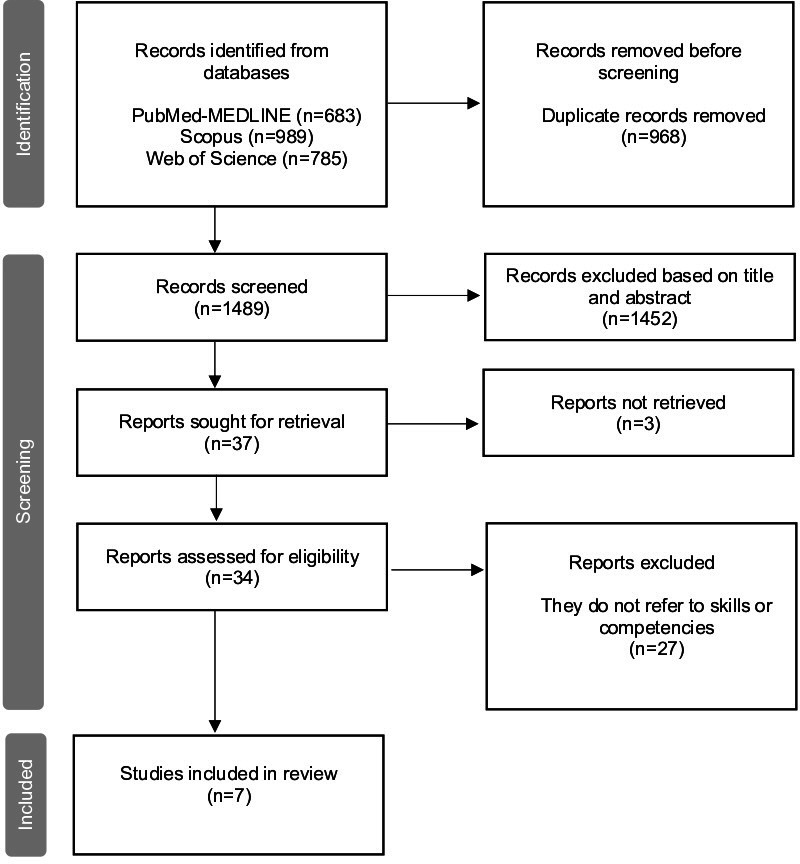
PRISMA flow diagram. PRISMA: Preferred Reporting Items for Systematic Reviews and Meta-Analyses.

The initial database searches yielded a total of 2457 articles (Figure 1). After deleting duplicates and screening titles and abstracts, 37 articles were selected for full-text review. Out of these, only 7 met all the inclusion criteria for this systematic review [23-29]. Each selected article specifically concentrated on the competencies essential for the integration of AI into routine clinical practice. A substantial number of the excluded articles (n=28) made reference to, but did not directly engage with, the competencies in question. Table S1 in Multimedia Appendix 1 delineates the characteristics of these studies and the principal competencies identified therein.

The review identified a diverse range of skills and competencies necessary for the effective implementation of AI in health care. These competencies were categorized into several key areas based on their frequency of appearance in the selected studies. The identified skills, definitions, and the number of studies that reported each are summarized in Table S2 in Multimedia Appendix 1.

### Identified Skills

The analysis of the selected studies highlights several key competencies deemed essential for the effective integration of AI into routine clinical practice.

AI fundamentals were identified as an essential competency by 86% of the studies [23-27,29]. This indicates a strong consensus on the importance of foundational AI knowledge for health care professionals. The studies emphasize that a solid understanding of AI principles is crucial for effectively implementing and using AI technologies in clinical settings. This foundational knowledge forms the bedrock upon which more advanced competencies are built, ensuring that professionals can confidently engage with AI tools and integrate them into their practice.

Ethical and legal considerations were emphasized by 71% of the studies [23-26,29]. The studies underscored the importance of having a solid knowledge base in various aspects of ethics, including patient privacy, data security, biases in algorithms, and transparency and explainability.

Data analysis and management skills were highlighted by 43% of the studies [24,26,28]. These studies emphasize several secondary skills crucial for effective data handling. For instance, McCoy et al [26] stress the importance of data acquisition, cleaning, and visualization as foundational steps in preparing data for AI applications. Singh et al [24] underline the need for robust data management practices to ensure the integrity and reliability of AI outputs. Wiljer and Hakim [28] highlight the necessity of developing capabilities in data governance, which includes the secure storage and regulatory compliance of health care data.

Communication and teamwork were identified as important competencies by 43% of the studies [23,25,27]. These studies underscore the critical need for effective communication skills to convey complex AI-related information to both colleagues and patients. Çalışkan et al [23] emphasize the role of interdisciplinary teamwork in the development and implementation of AI applications, highlighting the necessity for seamless collaboration between health care professionals and AI experts. Liaw et al [25] point out the importance of clear and empathetic communication with patients regarding the use of AI in their care, ensuring transparency and maintaining trust. Sujan et al [27] stress the need for health care teams to work cohesively to monitor and supervise AI systems, ensuring their safe and ethical use.

Evaluation of AI tools was mentioned by 43% of the studies [25,26,29]. These studies highlight several essential secondary skills necessary for the rigorous assessment of AI technologies. Liaw et al [25] emphasize the importance of understanding evidence-based evaluation methods to critically assess the performance and utility of AI tools in clinical practice. McCoy et al [26] discuss the need for health care professionals to be proficient in performing critical evaluations, including assessing the accuracy, reliability, and validity of AI algorithms. Sapci and Sapci [29] stress the significance of ongoing evaluation and monitoring of AI tools to ensure they consistently meet clinical standards and enhance patient outcomes.

Some investigations highlight more technical abilities, such as programming [26], or a deeper mathematical acumen [24,28]. These skills, while crucial, were not as frequently cited as the previously mentioned competencies. Conversely, a capability deemed highly significant across the literature is the aptitude for evaluating AI tools to ascertain their quality and rationalize their application, in addition to scrutinizing potential biases and limitations [24,25].

**Figure 2.**
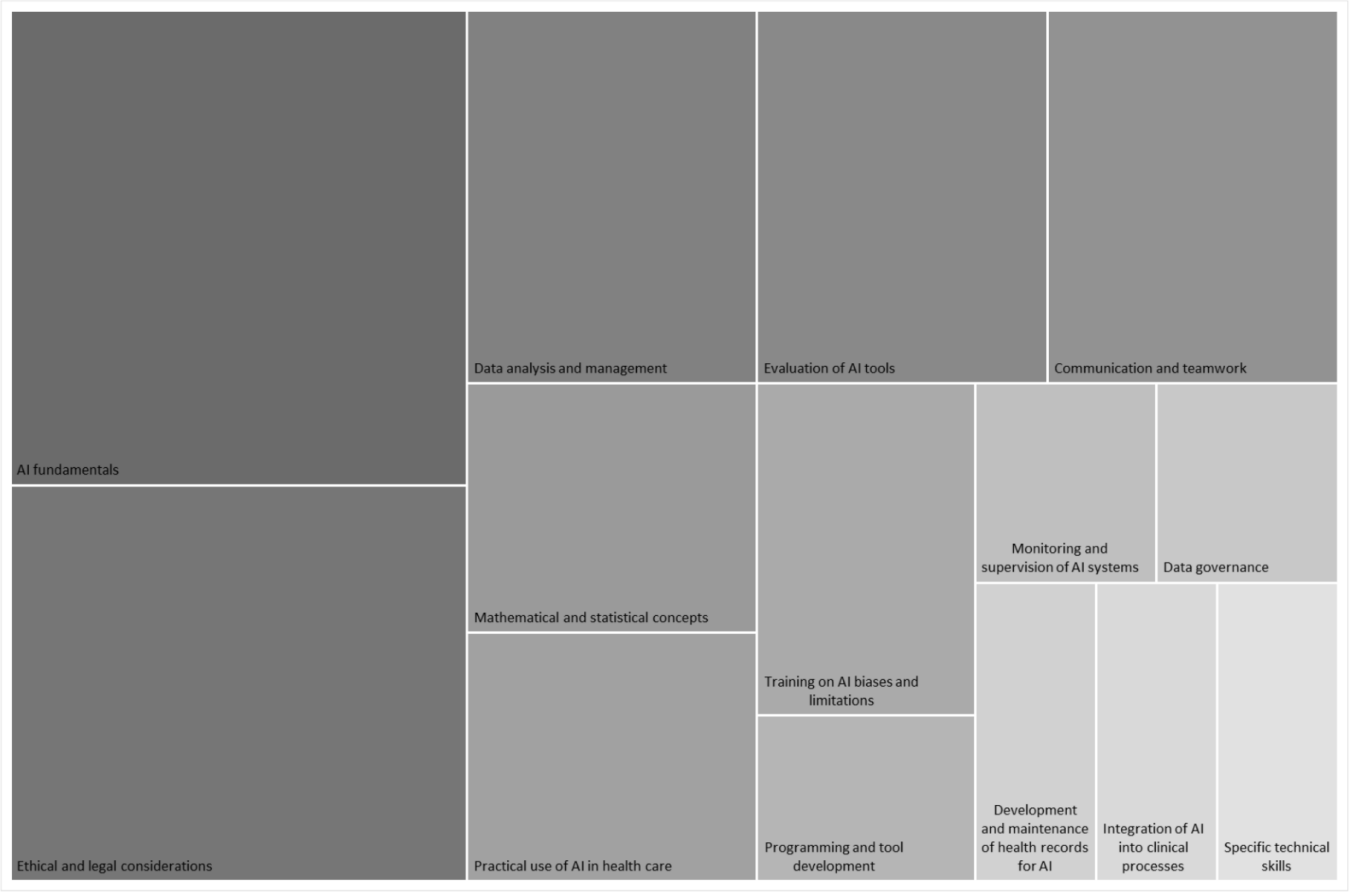
Distribution of identified competencies. AI: artificial intelligence.

Figure 2 presents a mosaic plot displaying the distribution of identified competencies based on the percentage of studies that reported each skill. The size of each tile corresponds to the proportion of studies mentioning that competency, providing a visual representation of the relative importance of each skill across the reviewed literature.

## Discussion

### General Aspects

This review underscores the critical need for health care professionals to acquire competencies related to the effective use of AI in clinical practice. Five key areas of competence were identified as essential for AI integration in clinical practice: AI fundamentals, ethical and legal considerations, data analysis and management, communication and teamwork, and the evaluation of AI tools. These competencies are essential to ensure the safe and reliable integration of AI technologies into routine health care workflows. Furthermore, ethical and legal considerations, such as data security and transparency, are crucial for building trust in AI-driven decisions (Table S2 in Multimedia Appendix 1).

In line with our findings, recent studies have emphasized the growing need to define and standardize AI competencies within clinical settings. A 2022 exploratory review [30] highlighted this gap, calling for more structured educational programs to support the integration of AI into health care practice. Additionally, a survey among nursing staff [31] indicated that most professionals had acquired their AI skills independently, further reinforcing the need for formalized training curricula.

### Skills

#### AI Fundamentals

The consensus across the literature highlights the essential nature of AI fundamentals [23-27,29]. This foundational knowledge is crucial for health care professionals to effectively implement and use AI technologies, providing the bedrock upon which more advanced competencies are built. AI fundamentals encompass a basic understanding of machine learning, neural networks, and deep learning. While health care professionals are not expected to have deep expertise in these technical areas, a solid grasp of how AI models function—how they are trained, how they process data, and how they support clinical decision-making—is necessary. This understanding allows professionals to effectively integrate AI into their workflows and ensure the appropriate use of AI-driven tools in patient care.

A key competency is the ability to critically assess AI algorithms, identify biases, and interpret the results. Studies, such as those by Faes et al [32], provide guidelines to help clinicians evaluate AI outputs without needing advanced computational skills, allowing them to focus on patient care. For example, clinicians should recognize when AI models may be limited by biased data or poor generalizability. Similar to using ultrasound technology—where clinicians do not fully understand the physics but can accurately interpret images—the same principle applies to AI. Health care professionals must develop the skills to interpret AI-generated information and effectively communicate it to patients, fostering trust and transparency in AI-driven care [33].

#### Ethical and Legal Considerations

Ethical and legal considerations were emphasized in 71% of the reviewed studies [23-26,29], highlighting the importance of health care professionals understanding and adhering to the ethical principles and legal frameworks governing the use of AI in clinical practice. These considerations include ensuring patient privacy, data security, and compliance with relevant regulations, which are critical for maintaining the trust and safety of patients.

Stöger et al [34] emphasized the crucial role of managing high-quality data and the risks associated with its mismanagement, underscoring both the ethical and legal implications for health care professionals using AI systems. In this context, health care providers must be familiar with the laws and regulations governing AI to ensure that the technology is implemented in compliance with legal and ethical standards.

In the European Union, the recent approval of the AI Act introduces comprehensive regulations [35] aimed at safeguarding fundamental rights and democracy by controlling AI systems based on their potential risks and impact. The AI Act establishes stringent requirements for high-risk AI systems, including mandatory impact assessments and prohibitions on certain AI practices that threaten fundamental rights. For health care professionals, this underscores the need for a thorough understanding of these legal frameworks to ensure that AI is used responsibly and safely in clinical practice.

Moreover, the opacity of machine learning algorithms, often referred to as “black boxes” due to their complex and abstract problem-solving methods, represents a significant challenge in ensuring accountability and transparency in AI-driven health care solutions [36]. Although efforts to make these algorithms more interpretable are still developing [37], health care professionals must rely on regulated AI tools that meet established standards for safety and efficacy. Regulatory bodies such as the US Food and Drug Administration have already approved specific AI tools for medical applications [38], providing a level of assurance that these technologies meet rigorous safety and legal standards. This regulatory oversight is critical for fostering trust in AI among clinicians and patients, while also mitigating liability concerns for health care providers.

#### Data Analysis and Management

Data analysis and management were identified as key competencies in 43% of the reviewed studies [24,26,28], emphasizing the critical role these skills play in the effective implementation of AI in health care. This domain encompasses the ability to handle large volumes of health care data, including data acquisition, cleaning, visualization, and governance. Health care professionals need to be proficient in organizing and managing these datasets to ensure the integrity and reliability of AI outputs.

A core competency in this area includes the preparation of clean and structured datasets for AI models, as highlighted by McCoy et al [26], who stress the importance of data preparation steps such as cleaning and visualization to optimize AI performance. Singh et al [24] underline the need for robust data management practices to guarantee that the data used by AI systems is accurate, reliable, and compliant with health care regulations.

Wiljer and Hakim [28] further highlight the significance of data governance, which includes not only managing data but also ensuring its secure storage and adherence to regulatory requirements. Proficient data handling is crucial, as health care professionals are often the primary data generators. Their ability to manage and interpret large datasets ensures that AI methodologies are applied effectively in clinical practice, supporting both the accuracy of AI predictions and the quality of patient care [39].

#### Evaluate AI Tools

The ability to evaluate AI tools emphasises the need for health care professionals to critically assess the performance, accuracy, and reliability of AI technologies in clinical practice. Effective evaluation is closely tied to a foundational understanding of AI principles, which allows professionals to determine whether AI tools are suitable for their specific clinical settings.

A key competency in this domain involves assessing AI tools with the same rigor applied to new drugs, diagnostic tests, or treatment protocols. AI-based tools must undergo extensive testing for accuracy, generalizability, efficacy, and fairness to ensure they meet clinical standards [25]. This evaluation process is crucial for ensuring that AI technologies deliver reliable outcomes and can be integrated safely into health care workflows.

Additionally, health care professionals must develop skills for the ongoing evaluation and monitoring of AI tools to detect any performance shifts or biases that may arise over time [40]. Given the continuous learning nature of AI systems, regular reassessment is necessary to maintain their reliability across diverse patient populations and clinical scenarios.

#### Communication and Teamwork

Communication and teamwork were identified as key competencies in 43% of the reviewed studies [23,25,27], focusing on the dual importance of effectively conveying AI-related insights to patients and facilitating collaboration among health care professionals. Health care providers must clearly explain AI-generated results, addressing patient concerns with empathy, ensuring transparency, and maintaining trust in the use of AI technologies in clinical care.

Equally important is the interdisciplinary collaboration required for integrating AI into clinical workflows. Health care professionals need to work closely with AI specialists, data scientists, and other colleagues to ensure the safe and effective use of AI tools. Studies such as those by Çalışkan et al [23] stress the necessity of seamless teamwork between health care providers and AI experts, while Liaw et al [25] underscore the need for clear and compassionate communication with patients, ensuring they understand how AI is applied in their care and the potential implications.

This competency involves not only understanding AI outputs but also contextualizing them for patients in a way that fosters trust and humanizes AI-assisted care. At the same time, health care teams must collaborate cohesively to monitor and manage AI systems. As AI streamlines routine tasks, health care providers will have more time for patient interaction, making effective and empathetic communication even more critical [25]. Ensuring that both patients and professional teams feel supported and understood is essential for the ethical and successful integration of AI in health care practice.

#### Competencies Based on Identified Skills

Based on the previously identified skills, and following an approach similar to that outlined by the Association of American Medical Colleges in 2021 [41], we propose a set of competencies that health care professionals should acquire to effectively integrate AI into clinical practice. These competencies, derived from the literature reviewed, encompass the key domains and provide a framework to guide educational programs and ongoing training in AI-driven tools in health care settings.

Table S3 in Multimedia Appendix 1 outlines the competencies for each skill, offering a structured approach to developing AI proficiency. This framework ensures that professionals not only grasp AI fundamentals but also apply these technologies ethically, manage health care data effectively, rigorously evaluate AI tools, and communicate insights to both patients and interdisciplinary teams. However, these competencies should be considered only a first proposal; this list may be modified depending on the specific profile of the training program, on foreseeable technological developments, as well as on the evaluation of the academic results of the new curricula.

### AI in Health Care Training

Recent data from Rock Health, a venture capital firm specializing in digital health, have underscored an exponential increase in investments directed toward digital health enterprises and related technologies [42]. This trend distinctly signals an evolving health care landscape, increasingly reliant on novel technologies, thereby accentuating the imperative for health care professionals to proficiently integrate such advancements into their clinical practices [43].

Despite widespread agreement on the necessity for comprehensive AI training from the outset of medical education, there is a lack of consensus on the specific content and approach of such training [15]. The discussion around this issue is abundant, yet concrete resolutions are rare. The findings of this review aim to provide an approach to the possible competencies required, offering a structured framework for developing comprehensive AI training programs for health care professionals.

Integrating AI education into health care curricula presents several challenges due to the variability and lack of standardization of required competencies. To address this need, we propose general guidelines for the development of essential AI competencies for health care professionals. These guidelines provide a reference framework that educators and course coordinators can use to design training programs, assess student progress, and establish performance criteria. For example, health care professionals should acquire the ability to assess the quality of algorithms and their interpretations, as well as identify and mitigate potential biases. This approach facilitates the integration of AI into clinical practice and provides a clear guide for curriculum development and performance evaluation.

Augmenting the education and training of health care professionals is posited to elevate their confidence in using these tools. Although concerns persist regarding AI’s potential to supplant human roles, a more discerning view proposes that AI will primarily alleviate the burden of mundane tasks. This reallocation of time and resources is anticipated to enhance patient interactions and elevate the quality of health care services provided [39].

Several authors argue that the existing academic infrastructure is ill-equipped to incorporate AI education, citing time constraints and a lack of teaching expertise as significant obstacles. An alternative proposed involves the use of specific AI tools not only for clinical applications but also to elucidate the underlying algorithms, focusing on their practical use and ethical implications [44].

A solution for integrating AI education into health care curricula, addressing the shortage of instructors with expertise in clinical AI applications, involves leveraging established training programs from other institutions [45]. Programs by Stanford University [46] and Harvard University [47] serve as examples, providing access to high-quality educational content. These programs offer a comprehensive curriculum that covers essential AI concepts, practical applications, and ethical considerations, enabling health care professionals to gain a deep understanding of AI technologies.

The integration of AI into clinical practice is expected to augment, not replace, the roles of health care professionals. It calls for a workforce proficient in digital health and communication, capable of leveraging AI’s benefits while recognizing its limitations and ethical considerations [48]. This paradigm shift offers an opportunity to enhance patient care, delegating computational tasks to AI and focusing on the human aspects of health care delivery [37].

### Limitations

This systematic review encounters several challenges, primarily due to the limited availability of literature specifically addressing the competencies required for integrating AI into health care practice. The scarcity of targeted studies can be attributed to the nascent and rapidly evolving nature of AI applications in health care.

To mitigate this issue, the search criteria were broadened to include general terms related to AI in health care and competencies required by health care professionals. While necessary, this broadening may have introduced studies that do not exclusively focus on AI competencies, potentially affecting the homogeneity of the findings. The review also faced potential language bias, as it primarily focused on literature in English and Spanish. Pertinent studies in other languages might have been excluded. Despite rigorous and independent review processes, the selection could still be influenced by subjective interpretation of the inclusion and exclusion criteria.

Limiting the review to studies published after 2018 aimed to capture the most recent advancements, but this restriction might omit emerging research. Despite these efforts, there remains a clear knowledge gap in the specific skills required for effective AI use in health care. The literature predominantly addresses AI applications and benefits, but lacks detailed research on the precise skills health care professionals need. Current research is often fragmented and varies significantly in scope and depth. Few studies offer comprehensive models or curricula for AI competency training in health care. This inconsistency highlights the need for more robust research to identify essential AI skills and explore effective methods for integrating these skills into health care education and practice.

### Conclusions and Future Works

This systematic review has identified essential competencies for health care professionals to effectively integrate AI into clinical practice. The analysis reveals a consensus on the importance of five key areas: AI fundamentals, ethical and legal considerations, data analysis and management, communication and teamwork, and evaluation of AI tools. These competencies are crucial for leveraging AI technologies to enhance patient care and health care delivery. A consensus within the scholarly discourse suggests the necessity for health care professionals to attain proficiency in these domains to ensure the judicious application of AI tools, thereby accruing benefits for both patients and the health care ecosystem.

AI fundamentals form the backbone of necessary knowledge, enabling health care professionals to understand and use AI technologies effectively. Ethical and legal considerations ensure that AI applications adhere to patient privacy, data security, and transparency standards, maintaining trust and compliance within health care settings. Data analysis and management skills are vital for handling large datasets, ensuring accurate AI outputs, and supporting informed clinical decisions.

Communication and teamwork are also critical, facilitating the clear conveyance of AI-related information among health care professionals and to patients, thereby promoting transparency and trust. The ability to evaluate AI tools is essential for assessing the performance and reliability of AI technologies, ensuring they meet clinical standards and deliver safe, effective patient care.

Augmenting the education and training of health care professionals is posited to elevate their confidence in using these tools. Although concerns persist regarding AI’s potential to supplant human roles, a more discerning view proposes that AI will primarily alleviate the burden of mundane tasks. This reallocation of time and resources is anticipated to enhance patient interactions and elevate the quality of health care services provided.

In this context, the importance of communication skills becomes increasingly paramount. The introduction of AI tools is expected to afford health care professionals additional time per patient encounter, potentially heightening patient satisfaction and care quality.

The ambition extends beyond merely acquiring proficiency in disciplines ancillary to traditional health care paradigms. Considering the already intricate and comprehensive nature of health care education, particularly in medicine, the emphasis is placed on fostering an in-depth comprehension of AI’s functionalities, inherent biases, pragmatic utility, and cost-effectiveness compared to abstaining from AI applications.

The integration of AI into health care is indispensable for advancing patient care but requires a concerted effort to develop and standardize competencies among health care professionals. Regulatory oversight and enhanced educational frameworks are essential for overcoming existing barriers and leveraging AI’s full potential in clinical settings.

Despite the progress highlighted in this review, significant gaps remain in the literature, particularly concerning the specific educational frameworks and training programs needed to develop these competencies. Most existing research focuses on the potential applications and benefits of AI, with less emphasis on the precise skills required for effective implementation.

Future research should prioritize the development and validation of standardized AI competency frameworks tailored for health care professionals. These frameworks should cover technical skills, ethical and legal aspects, and data management practices. Collaborative efforts between academic institutions, health care organizations, and AI experts can create comprehensive training programs to address these competencies.

Additionally, longitudinal studies are necessary to evaluate the long-term effectiveness of AI training programs. Research should explore how health care professionals apply their AI training in clinical settings, assessing the impact on patient outcomes, clinical decision-making, and health care efficiency.

## Supplementary material

10.2196/58161 Multimedia Appendix 1 Additional tables.

10.2196/58161 Checklist 1 PRISMA checklist.

## Abbreviations

AI: artificial intelligence
GRADE: Grading of Recommendations Assessment, Development, and Evaluation
PRISMA: Preferred Reporting Items for Systematic Reviews and Meta-Analyses
