# Supplementary material for: AI in the Health Sector: Systematic Review of Key Skills for Future Health Professionals
Source: JMIR Med Educ. 2025 Feb 5;11:e58161. doi: 10.2196/58161 (PMC11822726; doi:10.2196/58161)
Supplement: Multimedia Appendix 1 [file mededu-v11-e58161-s001.docx]

**Table S1.** Studies Included in the review.

| Title | Authors | Type of Study | Identified Skills |
| --- | --- | --- | --- |
| *Artificial intelligence in medical education curriculum: An e-Delphi study for competencies*[22] | Çalışkan SA, Demir K, Karaca O. | Delphi | Use of health data according to legal and ethical standards.  Acting according to ethical principles when using AI technologies.  Appropriate use of AI applications.  Maintenance of health records for AI processing.  Use of information from AI applications along with professional knowledge.  Assessment of AI use for educational, service, and research purposes.  Explanation of how AI applications in health offer solutions to specific problems.  Organization of workflow in accordance with AI work logic.  Effective and efficient use of AI technologies in health service delivery.  Definition of basic data science concepts.  Keeping up with current developments and literature on the use of AI technologies in health.  Teamwork with experts in the development of AI applications.  Accessing, evaluating, using, sharing, and creating new information through information and communication technologies.  Following legal regulations regarding the use of AI technologies in health.  Expressing the importance of data collection, analysis, evaluation, and security for the development of AI applications in health.  Anticipating opportunities and threats that AI technology may create.  Differentiating between functions and features of AI-related tools and applications.  Proper analysis of data obtained by AI in health.  Definition of basic concepts and terminology of AI.  Definition of basic statistical concepts.  Decision-making regarding the use of AI technologies in health.  Explanation of how doctors' knowledge and experiences can be used in developing AI applications.  Choosing the appropriate AI application for health problems. |
| *Current challenges and barriers to real-world artificial intelligence adoption for the healthcare system, provider, and the patient.*[23] | Singh RP, Hom GL, Abramoff MD, Campbell JP, Chiang MF, on behalf of the AAO Task Force on Artificial Intelligence | Perspective Analysis | AI fundamentals.  Mathematical concepts.  Data analysis and management.  Training on AI Biases and Limitations.  Ethical and Legal Issues. |
| *Competencies for the use of artificial intelligence in primary care* [24] | Liaw W, Kueper JK, Lin S, Bazemore A, Kakadiaris I. | Theoretical Review | AI fundamentals.  Legal regulations of AI tools.  Evidence evaluation of tools.  Skills in tool use and data capture.  Technical Use: Technical skills needed to efficiently operate AI-based tools.  Communicating with patients to inform about AI use. |
| *What do medical students actually need to know about artificial intelligence?*[25] | McCoy LG, Nagaraj S, Morgado F, Harish V, Das S, Celi LA. | Conceptual Review / Commentary | Understanding of AI applications in Health.  Skills in Data analysis, acquisition, cleaning, and visualization  Programming skills.  Critical evaluation of AI.  Conceptual understanding of AI and Clinical Data Science.  Ethical Considerations in AI.  Dual training in medicine and Informatics/Data Science. |
| *Stakeholder perceptions of the safety and assurance of artificial intelligence in healthcare*[26] | Sujan, Mark A.; White, Sean; Habli, Ibrahim; Reynolds, Nick | Semi-structured Interview | AI fundamentals.  Monitoring and supervision of AI systems.  Understanding and conveying information generated by AI.  Promoting trust in AI.  Maintaining essential clinical skills despite automation. |
| *Developing an Artificial Intelligence–Enabled Health Care Practice: Rewiring Health Care Professions for Better Care*[27] | David Wiljer, Zaki Hakim, | Commentary | Data governance principles.  Basic statistics and algorithmic decision-making.  Data visualization capabilities and storytelling.  Understanding how business or clinical processes will be altered by integrating artificial intelligence technologies into healthcare. |
| *Artificial Intelligence Education and Tools for Medical and Health Informatics Students: Systematic Review*[28] | Sapci A, Sapci H | Systematic Review | AI predictive techniques.  AI ethics.  Evaluation of AI tools. |

**Table S2.** Identified skills.

| Identified Skill | Number of Studies | Percentage of Studies (%) | Definition | Example |
| --- | --- | --- | --- | --- |
| AI fundamentals | 6 | 85.71% | Basic understanding of AI principles, including machine learning, neural networks, and deep learning, is necessary for healthcare professionals. While not requiring deep expertise, this foundational knowledge enables them to effectively apply AI tools in clinical practice | Understanding how AI models are trained and used to support clinical decisions |
| Ethical and legal considerations | 5 | 71.43% | Understanding the ethical implications of AI, including patient privacy, algorithmic bias, and data security | Ensuring that AI-driven decisions are explainable to patients and addressing the potential risks of bias in AI systems. Understanding legal frameworks |
| Data analysis and management | 3 | 42.86% | Skills related to handling healthcare data, including data acquisition, cleaning, visualization, and governance | Preparing clean and structured datasets for AI models, and ensuring compliance with regulations in health care data storage and management. Effective data handling ensures that AI outputs are accurate and reliable |
| Evaluation of AI tools | 3 | 42.86% | Ability to assess the performance, accuracy, and reliability of AI technologies in clinical practice. | Evaluating the accuracy, generalizability, and fairness of an AI-based diagnostic tool, ensuring it meets clinical standards. Understanding the need for ongoing evaluation of AI systems to detect inconsistencies or biases |
| Communication and teamwork | 3 | 42.86% | The ability to effectively communicate AI-related insights to patients, ensuring transparency and maintaining trust, while also collaborating with colleagues in interdisciplinary teams. | Clearly explaining AI-generated diagnostic results to patients, addressing their concerns, and ensuring that healthcare professionals work cohesively with AI experts and other team members to integrate AI tools into clinical workflows |
| Mathematical and statistical concepts | 2 | 28.57% | Basic understanding of mathematical foundations behind AI algorithms, including probability, statistics, and linear algebra | Using statistical methods to interpret AI predictions and ensuring their clinical validity |
| Practical use of AI in healthcare | 2 | 28.57% | Hands-on application of AI technologies in clinical environments | Implementing AI tools to assist in clinical decision-making, such as using AI algorithms to interpret medical images in radiology |
| Training on AI biases and limitations | 2 | 28.57% | Awareness and understanding of the potential biases and limitations of AI technologies | Recognizing how biased training data can lead to biased AI outcomes, and taking steps to minimize these risks in clinical settings |
| Programming and tool development | 1 | 14.29% | Ability to develop or modify AI tools through programming | Writing code to adapt an AI tool for a specific clinical application |
| Monitoring and supervision of AI systems | 1 | 14.29% | Continuous oversight of AI systems to ensure their appropriate use and performance | Regularly checking the performance of AI systems to ensure they meet clinical safety standards and function as expected |
| Data governance | 1 | 14.29% | Skills related to the secure handling, storage, and compliance of healthcare data used by AI | Implementing data governance strategies to ensure AI systems comply with laws such as GDPR in handling patient |
| Development and maintenance of health records for AI | 1 | 14.29% | Knowledge of structuring and maintaining electronic health records (EHRs) to optimize AI systems. | Organizing EHR data to ensure that it can be efficiently processed by AI tools to generate useful insights for clinical decision-making |
| Integration of AI into clinical processes | 1 | 14.29% | Ability to seamlessly integrate AI tools into clinical workflows | Introducing AI-based decision support systems into routine clinical practice to enhance diagnostic accuracy and efficiency |
| Specific technical skills | 1 | 14.29% | Technical proficiency in managing and deploying AI tools in clinical settings. | Configuring and troubleshooting AI systems to ensure they operate correctly and effectively in healthcare environments |

**Table S3.** Competencies for AI in health care across the five skills identified.

|  | Competencies | |
| --- | --- | --- |
| Identified Skill | **Recently Graduated Healthcare Professionals** | **Experienced Healthcare Professionals** |
| AI fundamentals | - Recognize how AI models are used to support decision-making in clinical settings - Understand basic principles of AI, including machine learning and neural networks - Know how AI models perform and how their training and validation is done. | - Evaluate the applicability and effectiveness of AI technologies in specific clinical environments. - Demonstrate a deeper understanding of AI limitations and actively mitigate potential biases in practice. - Provide guidance to colleagues on implementing AI tools within clinical workflows |
| Ethical and legal considerations | - Understand key ethical concerns, such as patient privacy, data security, and informed consent. - Comprehend the basic legal frameworks governing AI in healthcare - Identifies personal and institutional responsibilities arising from the use of AI-based tools in health care. | - Apply ethical principles in complex AI use cases, ensuring AI systems are deployed responsibly in clinical settings. - Act as a mentor in addressing legal and ethical challenges, ensuring accountability and transparency in AI-driven care. |
| Data analysis and management | - Handle healthcare datasets by performing data acquisition, cleaning, and visualization tasks in alignment with best practices. - Ensure data integrity in the use of AI, preparing structured data for clinical applications. - Understand the factors that influence data quality and how they affect the results of AI-based applications. | - Oversee large-scale data management, ensuring compliance with data governance regulations. - Act as a liaison between healthcare and data science teams to ensure seamless data handling. |
| Evaluation of AI tools | - Monitor AI systems to detect and report inconsistencies or biases. - Understand the potential impact of ethnicity, sex, gender or social determinants on the outcomes of AI-based tools. - Ensure AI tools meet clinical standards and are suitable for diverse patient populations. | - Lead the evaluation of AI tools, ensuring they meet rigorous standards of accuracy, efficacy, and fairness. - Identify the technological needs of AI tools, assessing the economic and energy cost of the different alternatives. - Continuously monitor AI systems and adapt tools to evolving clinical needs. |
| Communication and teamwork | - Communicate AI-generated outputs clearly to patients, ensuring transparency, empathy and trust. - Work effectively in interdisciplinary teams with AI experts and healthcare professionals to integrate AI tools. | - Lead teams in fostering effective collaboration between AI specialists and healthcare providers. - Design programmes to change roles, responsibilities and workflows associated with the adoption of new AI-based tools. - Mentor junior staff on AI communication techniques, ensuring patient understanding and acceptance. |
